# Is a Genetic Variant associated with Bipolar Disorder Frequent in People without Bipolar Disorder but with Characteristics of Hyperactivity and Novelty Seeking?

**DOI:** 10.2174/17450179-v19-e230419-2022-53

**Published:** 2023-05-15

**Authors:** Goce Kalcev, Alessandra Scano, Germano Orrù, Diego Primavera, Giulia Cossu, Antonio Egidio Nardi, Mauro Giovanni Carta

**Affiliations:** 1 Department of Innovation Sciences and Technology, University of Cagliari, Cagliari, Italy; 2 Department of Surgical Sciences, University of Cagliari, Cagliari, Italy; 3 Azienda Regionale della Salute (ARES, Sardegna), Medio Campidano, University of Cagliari, Cagliari, Italy; 4 Department of Medical Sciences and Public Health, University of Cagliari, Cagliari, Italy; 5 Laboratory Panic and Respiration, Institute of Psychiatry (Ipub), Federal University of Rio De Janeiro (Ufrj), Rio De Janeiro, Brazil

**Keywords:** Familiarity, Risk, Bipolar disorder, Hyperactivity, Novelty seeking, CACNA1C gene

## Abstract

**Objective:**

The objective is to verify whether a genetic condition associated with bipolar disorder (BD) is frequent in old adults adapted to their environment, without BD, but with aptitudes for hyperactivity and novelty seeking (H/NS).

**Methods:**

In this cross-sectional study, the study sample included healthy elderly people (40 participants, aged 60 or older) living in an urban area and recruited from a previous study on physical exercise and active aging, who were compared with 21 old adults with BD from the same area. The genetic methodology consisted of blood sampling, DNA extraction, real-time PCR jointly with FRET probes, and the SANGER sequencing method. The genetic variant RS1006737 of CACNA1C, found to be associated with bipolar disorder diagnosis, was investigated.

**Results:**

The frequency of the RS1006737 genetic variant in the study group (H/NS) is not higher than in the BD group and is statistically significantly higher than in all the control groups found in the literature. However, the familiarity for BD is higher in old adults with BD than in the H/NS sample without BD. The risk of BD in the family (also considering those without BD but with family members with BD) is not associated with the presence of the genetic variant examined.

**Conclusion:**

The study suggests that the gene examined is associated with characteristics of hyperactivity rather than just BD. Nevertheless, choosing to participate in an exercise program is an excessively general way to identify H/NS. The next step would be to identify the old adults with well-defined H/NS features with an adequate tool.

## 1 INTRODUCTION

It has been hypothesized that life in the modern world, especially in megacities, is likely to break normal biological rhythms. The current changed habits of work and nocturnal leisure activities, together with the stimuli due to light and noise pollution, do not match the typical pattern of alternating sleep-wakefulness and energy disposal fixed by history and millennia of adaptation of human beings [1]. According to some studies, we “are facing an evolutionary decoupling of habits and adaptive demands” [2]. In this new framework, people with characteristics of predisposition to have different biologic rhythms from what was considered normal in the previous era may have some adaptive resources [3]. According to this perspective, the characteristics of hyperactivity and relative decreased needs for sleep in the manic periods could represent pathological outcomes (due to stress and excessive usage) of the fundamental flexibility of the sleep-wake rhythms with some adaptive qualities. Mania could therefore be the pathological face of a characteristic which, if not excessively stressed, could have a positive side in modern life [4].

In exploring this hypothesis, we found that the frequency of episodes of non-pathological hypomania was double among the Sardinian migrants living in the megacities of South America compared to that of Sardinians residing in Sardinia, while the frequency of bipolar disorder (BD) and mood disorders was similar in the two groups. One of the push factors of migration of “volunteer” migrants is probably their aptitude for exploration and the search for novelty, which those who remain in their homeland don’t have [5, 6]. Although the results of this research are interesting, no study has so far focused on the potential adaptive significance of genetic variables associated with bipolar disorder.

This study aims to verify whether a genetic condition associated with bipolar disorder can also be frequent in perfectly adapted elderly people without bipolar disorders, characterized by an aptitude for hyperactivity and novelty seeking (H/NS). The characteristics of hyperactivity and novelty seeking are thought to be conferred in older adults by participation in and completion of a mild-moderate intensity physical activity program.

## 2 METHODS

### 2.1 Design of the Study

This is a cross-sectional study in which the frequency of the CACNA1C gene and the genetic variant RS1006737 was measured in a sample of old adults with hyperactivity / novelty-seeking features and compared with the frequency of the same gene and the same variant in a similar group of people with bipolar disorder from the same city and with the control samples identified in the literature [7-9]. This study also compared the familial risk for bipolar disorder in the study sample and the control group (consisting of individuals with a diagnosis of BD).

### 2.2 Participants and Recruitment

The study sample included older adults (40 participants) in the city area of Cagliari, Italy, who had previously been involved in a clinical trial on the efficacy of middle/moderate exercise [10, 11]. In order to be included in this trial, the participants, from both genders had to be 60 or older, to live at home and to be able to freely give informed consent. The trial’s exclusion criteria included severe medical conditions (mild chronic pathological conditions such as hypertension or diabetes were allowed) and inability to sustain a medium / moderate physical effort. The original sample did not include people diagnosed with lifetime bipolar spectrum disorder conditions, even if this was not an exclusion criterion. A control group of patients with a diagnosis of BD, aged 60 or older, from both genders, attending the Centro di Psichiatria di Consultazione e Psicosomatica of the University Hospital of Cagliari, Italy was also included in the study (21 participants).

### 2.3 Psychiatric Evaluation

Both groups underwent psychiatric evaluation, including psychiatric interviews (Mood Disorder Questionnaire (MDQ), the Biological Rhythms Interview of Assessment in Neuropsychiatry (BRIAN), the Patient’s Health Questionnaire (PHQ-9), the Health Survey Short Form (SF-12), and the Questionnaire for Adaptive Hyperactivity and Goal Achievement (AHGA)), together with the collection of personal and family history.

### 2.4 Genotyping

The genetic methodology consisted of 3 consecutive phases [12, 13]:

#### 2.4.1 Preanalytical Phase

A blood sampling of the 61 participants was carried out in the Molecular Biology Laboratory of the Department of Surgical Sciences of the University of Cagliari, Italy. Additionally, fluorescent hybridization probes and oligonucleotides for PCR (PRIMER) were developed to detect polymorphisms.

#### 2.4.2 Analytical Phase

Genomic DNA was extracted from the blood using Bosphore Viral DNA/RNA Extraction Spin Kit (Anatolia gene works, TURKEY). The concentration and the purity of the extract were measured with the NanoDrop spectrophotometric system (Thermo Scientific), which involved the use of hybridization fluorescent oligonucleotide probes (Hybridation probe, HP) based on the principle of FRET (Frequency Resonance Energy Transfer). The real-time PCR instrument, where the reaction took place, did only amplify the DNA fragment examined in the experiment, but it also allowed us to read the fluorescence emitted by the target DNA-ligated probes during the amplification reaction. Upon PCR completion, the Probe-DNA system was heated at constant velocity. In order to validate the specificity of this method, some samples were also analyzed using the Sanger sequencing Method.

#### 2.4.3 Post-analytical Phase

In this phase, the data from the obtained sequences were processed and compared through software. The sequencer's electropherogram enabled us to analyze the data with software such as Blast (https://blast.ncbi.nlm.nih.gov/Blast.cgi/) and Clustal W (https://www.ebi.ac.uk/Tools/msa/clustalo).

### 2.5 Ethical Considerations

The study protocol has been approved by the Comitato Etico Indipendente presso l’Azienda Mista Ospedaliero Universitaria di Cagliari (authorization of 11 July 2022, reference number NP / 2022 / 2893). All included subjects provided written informed consent.

### 2.6 CACNA1C Gene and Risk Genetic Variant RS1006737

The impact of calcium signalling on bipolar disorders has been studied for a long time. Particular genetic findings have shown that impaired calcium signalling is likely behind the pathophysiology of BD. Also, it has been found that there are modifications in calcium levels in the cerebrospinal fluid observed in mania [14]. One of the biggest cornerstones for impaired calcium signalling mediated by LTCCs (L-type calcium channels) is the CACNA1C gene, which encodes the LTCC Cav1.2 α1 subunit. It is known that the risk allele RS1006737 is the most frequently associated genetic risk for BD [15, 16]. The studies' findings indicate that the genetic mutation RS1006737 has been linked to alterations in brain circuitries, including elevated activity in the prefrontal region and hippocampi during executive function and emotional processing [17]. Other studies using brain imaging have also emphasized the role of the RS1006737 genotype in brain function, including greater amygdala activity during emotional processing, which can impair risk allele carriers' ability of facial emotion recognition [18-20].

### 2.7 Data Analysis

Chi-Square values, P-values, Odds ratios (ORs), and 95% confidence intervals (95% CI) were calculated for the genetic variant RS1006737 regarding its frequency in the group of older adults with hyperactivity / novelty-seeking traits and another similar group of BD subjects. The same statistical measures were also applied when comparing with the control samples found in the literature and concerning the study sample’s and the bipolar disorder control group's family risk of developing BD.

## 3 RESULTS

Table **1** shows the association of the genetic variant (RS1006737) in older adults with bipolar disorders and old adults without bipolar disorders, but with hyperactivity and novelty-seeking features. The comparison profile of the presence of the genetic variant (RS1006737) in our study sample *versus* control samples identified in the literature studies [7-9] is shown in Table **2**. Following that, the presence of this variant in our study sample is statistically significantly higher than in all three control groups found in the literature.

Regarding familiarity of bipolar disorder, Table **3** shows the frequency of family history for bipolar disorders in BD older subjects and novelty seekers separately, and Table **4** presents the frequency of the family history in old adults with or without BD, but with the presence of genetic variant (RS1006737) and old adults with or without BD and without the presence of the same genetic variant. A higher level of familiarity with BD is observed in old adults with BD than in the H/NS sample without BD. Also, the risk of BD in the family is not linked with the presence of the analysed genetic variant.

## 4 DISCUSSION

According to the findings of our study, the frequency of the RS1006737 genetic variant in the study group (H/NS) is not higher than in the BD group (Table **1**) and is statistically significantly higher than in all the control groups of three studies found in the literature [7-9] (Table **2**). For example, the first study showed a significant association in the Pakistan population for a heterozygous genotype with BD, with 34.2% of control individuals and 50.8% of cases [7]. In the second study, 68 subjects out of 124 control subjects were found to have the genetic variant (17 with a homozygous and 51 with a heterozygous profile) [8]. As reported by the results of the third study, 22 control individuals out of 50 had the RS1006737 genetic variant (3 homozygous and 19 heterozygous) [9].

However, the familiarity for BD is higher in old adults with BD than in the H/NS sample without BD (Table **3**). The risk of BD in the family (also considering those without BD, but with family members with BD) is not associated with the presence of the examined genetic variant (Table **4**). Several explanations for this outcome are possible: I) the gene may not be associated with the disorder but with some related variable; II) it would be useful to divide the sample without bipolar disorder (H/NS) into two groups: those who actually have a profile of hyperactivity and novelty seeking and those who don't have it. The CACNA1C gene that has always been associated with the risk of BD is probably associated with hyperactivity, which might be a determining factor. In other words, there is the possibility that a genetic characteristic considered a “weakness” eventually represents an advantage when used appropriately. The presence of hypеthymic temperament traits in conjunction with a bipolar genetic variant should not be interpreted as a subject's vulnerability. On the contrary, given today's rapid changes and modern lifestyle, if used correctly, they can represent a huge advantage in developing one's own potential, and, thus, for society.

The approach of the recruitment of the sample of older adults with hyperactivity and novelty seeking but without bipolar disorders needs to be considered. This group, that was recruited through public notices, consisted of participants, particularly the elderly ones, in good health and/or with minor health conditions (diabetes, hypertension, *etc.*), and who were able to perform moderate physical activities and did not have any lifetime mood or other mental health disorders. On the contrary, the participants of the other group suffered from bipolar disorders.

It is well known that this mental disease has a strong genetic component. As reported by Akiskal *et al.*, the underlying trait being passed on is not the BD syndrome but alterations in temperament [21]. Taking in consideration the results of one of his studies, certain dimensions of temperament are transmitted in families as quantitative traits that are part of a broader bipolar spectrum. For instance, the scores on the hyperthymic scale were significantly higher in controls (healthy subjects without any psychiatric diagnosis and having no family history of mental disorders), than in the BD group, and unaffected relatives [21]. Hence, the more extreme the temperamental variation, the greater the risk an individual has of developing BD [22, 23]. Both BD and temperament arise from a biological dysregulation of mood, which indicates that temperament is the closest and most direct effect of biological modification. In genetic terms, this suggests a higher penetrance of the trait and may ultimately certify to be a more effective and genetically potent BD phenotype [24-26], even if an appropriate definition for the BD phenotype has still to be chosen. A broad variety of mood-related traits are observed in the families of bipolar probands, indicating a complex association between genotype and phenotype [27, 28]. The genetic features are not always connected with a higher risk of developing the disease, but they could also have an adaptive implication in some backgrounds. Particular interest has been sparked by the interactions between genetic heritage and environmental factors in the areas of neurogenesis and the Hypothalamic-Pituitary-Adrenal (HPA) axis development [29]. Urban life's shifting or accelerating rhythms may have an impact on older adults' HPA axes. As a result, living in cities increases the risk of mental illness and mood disorders. Thus, individuals with hyperthymic temperaments and personalities (explorers and novelty seekers) may be able to adjust to new situations [30-32]. On the other hand, we should not forget the fact that the prevalence of bipolar disorders has increased in urban areas in the last decades, where the severity of the disease directly relates to exposure to modern living conditions and the environment [33, 34].

It is worth pointing out a few obvious limitations in our study. Firstly, the study has an exploratory and preliminary character, and its results need to be confirmed. Another limitation lies in the characteristics of the sample. As previously mentioned, most participants were recruited through the Active Aging study that focused on different physical and cultural activities. However, the consequent high percentage of people with the gene variant RS1006737 found in our study sample (much higher than anticipated in the general population, based on previous literature descriptions), shows that this is a non-representative sample of the general population and that hyperactivity is unbalanced.

## CONCLUSION

The study suggests that the gene examined is associated with characteristics of hyperactivity rather than just BD. Nevertheless, choosing to participate in an exercise program is an excessively general way to identify H/NS. The next step would be to identify the old adults with well-defined H/NS features with an adequate tool.

## Figures and Tables

**Table 1:** Gen+ Variant (RS1006737) in old adults with BD and old adults without BD, but with the features of H/NS.

Groups	Gen+ Variant (RS1006737) (AA/AG)	Gen- Variant (RS1006737) (GG)	Total	χ2	P	OR	CI 95%
Old Adults Without Bipolar Disorder, but novelty seekers (study sample)	25 (62.5%)	15 (37.5%)	40	Pivot	-	-	-
Control Group 1 [7]	41 (34.2%)	79 (65.8%)	120	9.938	0.002	3.28	1.5-6.7
Control Group 2 [8]	68 (54.84)	56 (46.67%)	124	0.723	0.395	1.37	0.6-2.8
Control Group 3 [9]	22 (44%)	28 (56%)	50	0.308	0.081	2.12	0.9-4.9
Pooled 3 Control Groups	131(44.55)	163	294	4.553	0.033	2.12	1.1-4.1

**Table 2:** Gen+ Variant (RS1006737) in old adults without BD and features of H/NS (present sample) and control groups found in literature.

Groups	With Family History of BD	Without Family History of BD	Total	χ2	P	OR +H/NS-BD	CI 95%
Old Adults with Bipolar Disorder	12	9	21	13.652	0.0001	9.33	2.6-33.4
Old Adults Without Bipolar Disorder (Novelty seekers)	5	35	40	-	-	-	-

**Table 3:** Family history for BD in old adults with BD and old adults without BD, but with the features of H/NS.

Groups	With Variant (RS1006737)	Without Variant (RS1006737)	Total	χ2	P	OR +H/NS-BD	CI 95%
Old Adults with Family History of BD	13	4	17	1.240	0.266	2.04	0.6-7.3
Old Adults Without Family History of BD	27	17	44	-	-	-	-

**Table 4:** Family history for BD in old adults with or without BD but with the presence of genetic variant (RS1006737) and old adults with or without BD without presence of genetic variant (RS1006737).

Groups	Gen+ Variant (RS1006737)	Gen- Variant (RS1006737)	Total	χ2	P	OR BD	CI 95%
Old Adults with Bipolar Disorder	15 (71%)	6	21	0.486	P=0.487	1.50	0.48-4.70
Old Adults Without Bipolar Disorder (Novelty seekers)	25 (62.5%)	15	40	-	-	-	-

## Data Availability

The dataset that supports the results and findings of this research are available from the corresponding author [G.K] upon reasonable request.

## References

[1] Carta M.G., Preti A., Akiskal H.S. (2018). Coping with the new era: Noise and light pollution, hperactivity and steroid hormones. Towards an evolutionary view of bipolar disorders.. Clin. Pract. Epidemiol. Ment. Health.

[2] Carta M.G., Colom F., Erfurth A., Fornaro M., Grunze H., Hantouche E., Nardi A.E., Preti A., Vieta E., Karam E. (2021). In memory of hagop akiskal.. Clin. Pract. Epidemiol. Ment. Health.

[3] Nardi A.E. (2018). Towards an evolutionary view of bipolar disorders.. Clin. Pract. Epidemiol. Ment. Health.

[4] Carta M.G., Perra A., Atzeni M., D’Oca S., Moro M.F., Kurotschka P.K., Moro D., Sancassiani F., Minerba L., Brasesco M.V., Mausel G., Nardi A.E., Tondo L. (2017). An evolutionary approach to mania studying Sardinian immigrants to Argentina.. Rev. Bras. Psiquiatr..

[5] Carta M.G., D’Oca S., Atzeni M., Perra A., Moro M.F., Sancassiani F., Mausel G., Nardi A.E., Minerba L., Brasesco V. (2016). Quality of life of sardinian immigrants in buenos aires and of people living in Italy and Sardinia: Does the kind of care have a role for people with depression?. Clin. Pract. Epidemiol. Ment. Health.

[6] Tost H., Champagne F.A., Meyer-Lindenberg A. (2015). Environmental influence in the brain, human welfare and mental health.. Nat. Neurosci..

[7] Khalid M., Driessen T.M., Lee J.S., Tejwani L., Rasool A., Saqlain M., Shiaq P.A., Hanif M., Nawaz A., DeWan A.T., Raja G.K., Lim J. (2018). Association of CACNA1C with bipolar disorder among the Pakistani population.. Gene.

[8] Mallas E., Carletti F., Chaddock C.A., Shergill S., Woolley J., Picchioni M.M., McDonald C., Toulopoulou T., Kravariti E., Kalidindi S., Bramon E., Murray R., Barker G.J., Prata D.P. (2017). The impact of CACNA1C gene, and its epistasis with ZNF804A, on white matter microstructure in health, schizophrenia and bipolar disorder.. Genes Brain Behav..

[9] Perrier E., Pompei F., Ruberto G., Vassos E., Collier D., Frangou S. (2011). Initial evidence for the role of CACNA1C on subcortical brain morphology in patients with bipolar disorder.. Eur. Psychiatry.

[10] Carta M.G., Cossu G., Pintus E., Zoccheddu R., Callia O., Conti G., Pintus M., Gonzalez C.I.A., Massidda M.V., Mura G., Sardu C., Contu P., Minerba L., Demontis R., Pau M., Finco G., Cocco E., Penna M.P., Orrù G., Kalcev G., Cabras F., Lorrai S., Loviselli A., Velluzzi F., Monticone M., Cacace E., Musu M., Rongioletti F., Cauli A., Ruggiero V., Scano A., Crisafulli A., Cosentino S., Atzori L., Massa E., Mela Q., Fortin D., Migliaccio G., Nardi A.E., Angermeyer M., Preti A. (2021). Active elderly and health—can moderate exercise improve health and wellbeing in older adults? Protocol for a randomized controlled trial.. Trials.

[11] Carta M.G., Cossu G., Pintus E., Zaccheddu R., Callia O., Conti G., Pintus M., Aviles Gonzalez C.I., Massidda M.V., Mura G., Sardu C., Contu P., Minerba L., Demontis R., Pau M., Finco G., Cocco E., Penna M.P., Orr G., Kalcev G., Cabras F., Lorrai S., Loviselli A., Velluzzi F., Monticone M., Cacace E., Musu M., Rongioletti F., Cauli A., Ruggiero V., Scano A., Crisafulli A., Cosentino S., Atzori L., Massa E., Mela Q., Fortin D., Migliaccio G., Machado S., Romano F., Preti A. (2021). Moderate exercise improves cognitive function in healthy elderly people: Results of a randomized controlled trial.. Clin. Pract. Epidemiol. Ment. Health.

[12] Orrù G., Carta M.G., Bramanti A. (2018). Design of FRET Probes for SNP RS1006737, Related to Mood Disorder.. Clin. Pract. Epidemiol. Ment. Health.

[13] Orrù G., Carta M.G. (2018). Genetic variants involved in bipolar disorder, a rough road ahead.. Clin. Pract. Epidemiol. Ment. Health.

[14] Kalcev G., Preti A., Scano A., Orrù G., Carta M.G. (2021). Insight into susceptibility genes associated with bipolar disorder: A systematic review.. Eur. Rev. Med. Pharmacol. Sci..

[15] Piras M., Scano A., Orrù G., Preti A., Marchese C., Kalcev G. (2021). Can an investigation of a single gene be effective in differentiating certain features of the bipolar disorder profile?. Clin. Pract. Epidemiol. Ment. Health.

[16] Szczepankiewicz A. (2013). Evidence for single nucleotide polymorphisms and their association with bipolar disorder.. Neuropsychiatr. Dis. Treat..

[17] Bigos K.L., Mattay V.S., Callicott J.H., Straub R.E., Vakkalanka R., Kolachana B., Hyde T.M., Lipska B.K., Kleinman J.E., Weinberger D.R. (2010). Genetic variation in CACNA1C affects brain circuitries related to mental illness.. Arch. Gen. Psychiatry.

[18] Wessa M., Linke J., Witt S.H., Nieratschker V., Esslinger C., Kirsch P., Grimm O., Hennerici M.G., Gass A., King A.V., Rietschel M. (2010). The CACNA1C risk variant for bipolar disorder influences limbic activity.. Mol. Psychiatry.

[19] Jogia J., Ruberto G., Lelli-Chiesa G., Vassos E., Maierú M., Tatarelli R., Girardi P., Collier D., Frangou S. (2011). The impact of the CACNA1C gene polymorphism on frontolimbic function in bipolar disorder.. Mol. Psychiatry.

[20] Nieratschker V., Brückmann C., Plewnia C. (2015). CACNA1C risk variant affects facial emotion recognition in healthy individuals.. Sci. Rep..

[21] Evans L., Akiskal H.S., Keck P.E., McElroy S.L., Sadovnick A.D., Remick R.A., Kelsoe J.R. (2005). Familiality of temperament in bipolar disorder: Support for a genetic spectrum.. J. Affect. Disord..

[22] Price R.A., Kidd K.K., Pauls D.L., Gershon E.S., Prusoff B.A., Weissman M.M., Goldin L.R. (1985). Multiple threshold models for the affective disorders: The Yale-NIMH collaborative family study.. J. Psychiatr. Res..

[23] Akiskal H.S. (2005). Searching for behavioral indicators of bipolar II in patients presenting with major depressive episodes: The “red sign,” the “rule of three” and other biographic signs of temperamental extravagance, activation and hypomania.. J. Affect. Disord..

[24] Akiskal H.S., Akiskal K., Allilaire J.F., Azorin J.M., Bourgeois M.L., Sechter D., Fraud J.P., Chatenêt-Duchêne L., Lancrenon S., Perugi G., Hantouche E.G. (2005). Validating affective temperaments in their subaffective and socially positive attributes: Psychometric, clinical and familial data from a French national study.. J. Affect. Disord..

[25] Cloninger C.R., Svrakic D.M., Przybeck T.R. (1993). A psychobiological model of temperament and character.. Arch. Gen. Psychiatry.

[26] Tavormina G. (2010). The temperaments and their role in early diagnosis of bipolar spectrum disorders.. Psychiatr. Danub..

[27] Akiskal H.S., Akiskal K.K., Lancrenon S., Hantouche E. (2006). Validating the soft bipolar spectrum in the French National EPIDEP Study: The prominence of BP-II 1/2.. J. Affect. Disord..

[28] Ghaemi S.N. (2013). Bipolar spectrum: A review of the concept and a vision for the future.. Psychiatry Investig..

[29] Carta M.G., Ouali U., Perra A., Ben Cheikh Ahmed A., Boe L., Aissa A., Lorrai S., Cossu G., Aresti A., Preti A., Nacef F. (2021). Living with bipolar disorder in the time of COVID-19: Biorhythms during the severe lockdown in Cagliari, Italy, and the moderate lockdown in Tunis, Tunisia.. Front. Psychiatry.

[30] Dias G.P., Cavegn N., Nix A., do Nascimento Bevilaqua M.C., Stangl D., Zainuddin M.S.A., Nardi A.E., Gardino P.F., Thuret S. (2012). The role of dietary polyphenols on adult hippocampal neurogenesis: Molecular mechanisms and behavioural effects on depression and anxiety.. Oxid. Med. Cell. Longev..

[31] McEwen B.S. (2013). The Brain on Stress.. Perspect. Psychol. Sci..

[32] Herman J.P., Cullinan W.E. (1997). Neurocircuitry of stress: Central control of the hypothalamo–pituitary–adrenocortical axis.. Trends Neurosci..

[33] Belvederi Murri M., Prestia D., Mondelli V., Pariante C., Patti S., Olivieri B., Arzani C., Masotti M., Respino M., Antonioli M., Vassallo L., Serafini G., Perna G., Pompili M., Amore M. (2016). The HPA axis in bipolar disorder: Systematic review and meta-analysis.. Psychoneuroendocrinology.

[34] Orrù G., Romano F., Scano A. (2020). The climate in the european union and the enlarged european region is a determinant of the COVID-19 case fatality ratio.. Biointerface Res. Appl. Chem..

